# A new species of deep-sea sponge-associated shrimp from the North-West Pacific (Decapoda, Stenopodidea, Spongicolidae)

**DOI:** 10.3897/zookeys.685.11341

**Published:** 2017-07-13

**Authors:** Peng Xu, Yadong Zhou, Chunsheng Wang

**Affiliations:** 1 Laboratory of Marine Ecosystem and Biogeochemistry, Second Institute of Oceanography, State Oceanic Administration, Hangzhou, 310012, China; 2 State Key Laboratory of Satellite Ocean Environment Dynamics, Second Institute of Oceanography, State Oceanic Administration, Hangzhou, 310012, China

**Keywords:** Hexactinellida, Magellan Seamount Chain, Spongicoloides, Weijia Guyot

## Abstract

A new species of the deep-sea spongicolid genus *Spongicoloides* Hansen, 1908 is described and illustrated based on material from the northwestern Pacific. *Spongicoloides weijiaensis* **sp. n.** was found inside a hexactinellid sponge, *Euplectella* sp., sampled by the Chinese manned submersible “Jiaolong” at depths of 2279 m near the Weijia Guyot, in the Magellan Seamount Chain. The new species can be distinguished from all congeneric species by several morphological features, involving gill formula, spination of the carapace, antennal scale, third pereiopod, telson and uropod, posteroventral teeth of the pleura, and dactyli of the fourth and fifth pereiopods. An identification key to the Pacific species of *Spongicoloides* is provided.

## Introduction

The stenopodidean shrimp family Spongicolidae is a relatively small group of marine decapod crustaceans. Based on its gill formula and external morphological features, the genus *Spongicoloides* Hansen, 1908 represents the most derived group among spongicolid genera (Saito and Takeda 2003). Although de Saint Laurent and Cleva (1981) synonymized *Spongiocaris* Bruce & Baba, 1973 with *Spongicoloides* and emended the generic diagnosis of *Spongicoloides*, Holthuis (1993) maintained *Spongiocaris* as a valid genus and his classification was followed by subsequent workers (e.g., Berggren 1993, Ortiz et al. 2007, Komai and Saito 2006, Saito and Komai 2008, Goy 2015, González et al. 2016, Komai et al. 2016). The presence of the exopod on the second maxilliped is the major characteristic used to distinguish *Spongiocaris* from *Spongicoloides*, so Saito (2008) transferred *Spongicoloides koehleri* (Caullery, 1896) to *Spongiocaris*. Chen et al. (2016) attempted to solve the controversial higher taxonomy of infraorder Stenopodidea using sequence data from both mitochondrial and nuclear genes. All their findings indicated that the morphological characters currently adopted to define genera are mostly invalid and substantial taxonomic revisions are required. Although they suggested that the genera such as *Spongicola* de Haan, 1844, *Spongicoloides* and *Spongiocaris* need to be redefined and revised with particular caution in the future, we accept *Spongicoloides* as a valid genus for the time being in this study. Eight species are currently known in *Spongicoloides*, and the loss of gills and the loss of spination on some body parts (carapace, pereiopods, pleon and tail fan) are thought to be secondarily derived in relation to the shrimps’ highly specialized sponge-dwelling habits.

**Figure 1.**
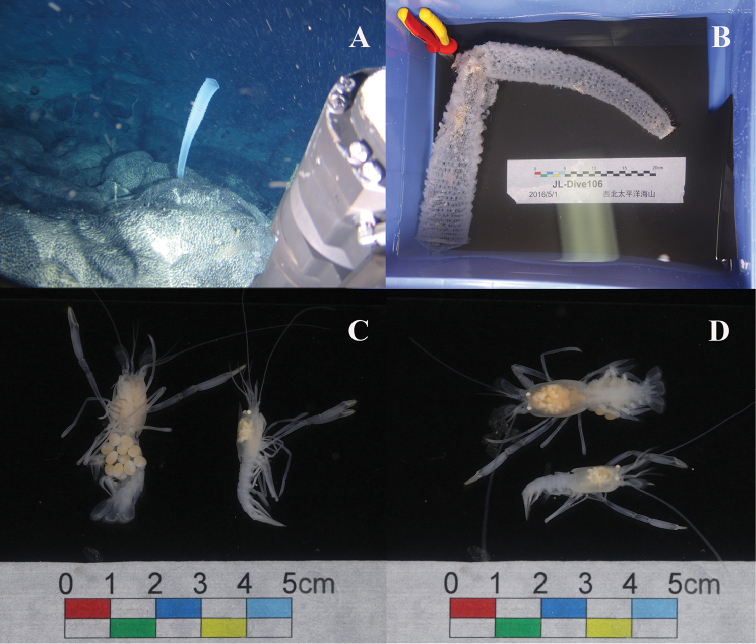
Hexactinellid sponge, *Euplectella* sp., *in situ* (**A**) and shortly after collection (**B**) **C, D** *Spongicoloides weijiaensis* sp. n., ovigerous female and male shortly after extraction from the host sponge.

In May of 2016, during Dive 106 of the Chinese manned submersible “Jiaolong”, one specimen of a hexactinellid sponge, *Euplectella* sp. (Fig. 1A, B), was sampled at a depth of 2279 m near Weijia Guyot, part of the Magellan Seamount Chain in the northwestern Pacific. On board of the vessel, a pair of spongicolid shrimps (Fig. 1C, D) was found inside this sponge. Since the absence of exopods on the second and third maxillipeds is one of the most important characteristics of *Spongicoloides*, the shrimps were assigned to that genus. After a careful comparison with all congeneric species, they were confirmed to be a new species, which is described and illustrated in this study, representing the ninth species of the genus.

## Materials and methods

The type specimens were preserved in 80% ethanol and deposited in the Sample Repository of Second Institute of Oceanography (SRSIO), State Oceanic Administration, Hangzhou, China.

Marginal spines of the telson are counted as: spines on the lateral margin; spine on the posterolateral angle, at the termination point of the dorsolateral carina; and spines on the posterior margin. Postorbital carapace length (in mm) is abbreviated as **cl** in the text. In the laboratory, photographs were taken using a dissecting microscope (Zeiss Discovery V20) equipped with a camera (AxioCam ICc5). Line drawings were made with the aid of a drawing tube mounted on a LEICA M205 C stereomicroscope. Setae have been omitted from illustrations for clarity.

## Systematics

### Family Spongicolidae Schram, 1986

#### Genus *Spongicoloides* Hansen, 1908

##### Spongicoloides weijiaensis sp. n.

Taxon classificationAnimaliaDecapodaSpongicolidae

http://zoobank.org/7BB95D26-0799-4078-B8F2-8C0AB4883D79

Figs 2 , 3 , 4 , 5

###### Material examined.

Holotype: ovigerous female, cl 11.1 mm, 13°01.01'N, 156°56.71'E, near Weijia Guyot, Magellan Seamount Chain, North West Pacific, depth: 2279 m, associated with hexactinellid sponge, coll. team of “Jiaolong” submersible, 1 May 2016, sample 37I-JL106-1, SRSIO16050001.

Paratype: male, cl 9.3 mm, same collection data as for holotype, sample 37I-JL106-2, SRSIO16050002.

###### Diagnosis.

Rostrum nearly horizontal, reaching to distal margin of basal article of antennular peduncle; rostral base triangular in dorsal view, each ventrolateral ridge armed with a minute spine. Carapace with distinct cervical groove; anterolateral margin with branchiostegal and pterygostomial spines, and several spinules situated posterior to them; postorbital region armed with one short longitudinal row of spinules; groups of similar spinules also present on posterior portion of cervical groove and rostrum. Second to fourth pleura each with one articular knob; first to third pleura broadly rounded and fourth to sixth pleura each with several posteroventral teeth. Telson quadrangular, with two conspicuous dorsolateral carinae each bearing 7–10 posteriorly directed spines. Eye devoid of dark pigment; eyestalk armed with minute spines. Lateral margin of antennal scale slightly concave, armed with 10–12 spines. Fixed finger of third pereiopod without row of small teeth on distoventral margin; ischium of third pereiopod with one row of 2–4 small teeth on flexor margin. Dactyli of fourth and fifth pereiopods biunguiculate primarily, bearing several much smaller accessory teeth arising from bases of ventral and dorsal ungues.

###### Description of holotype female.

**Figure 2.**
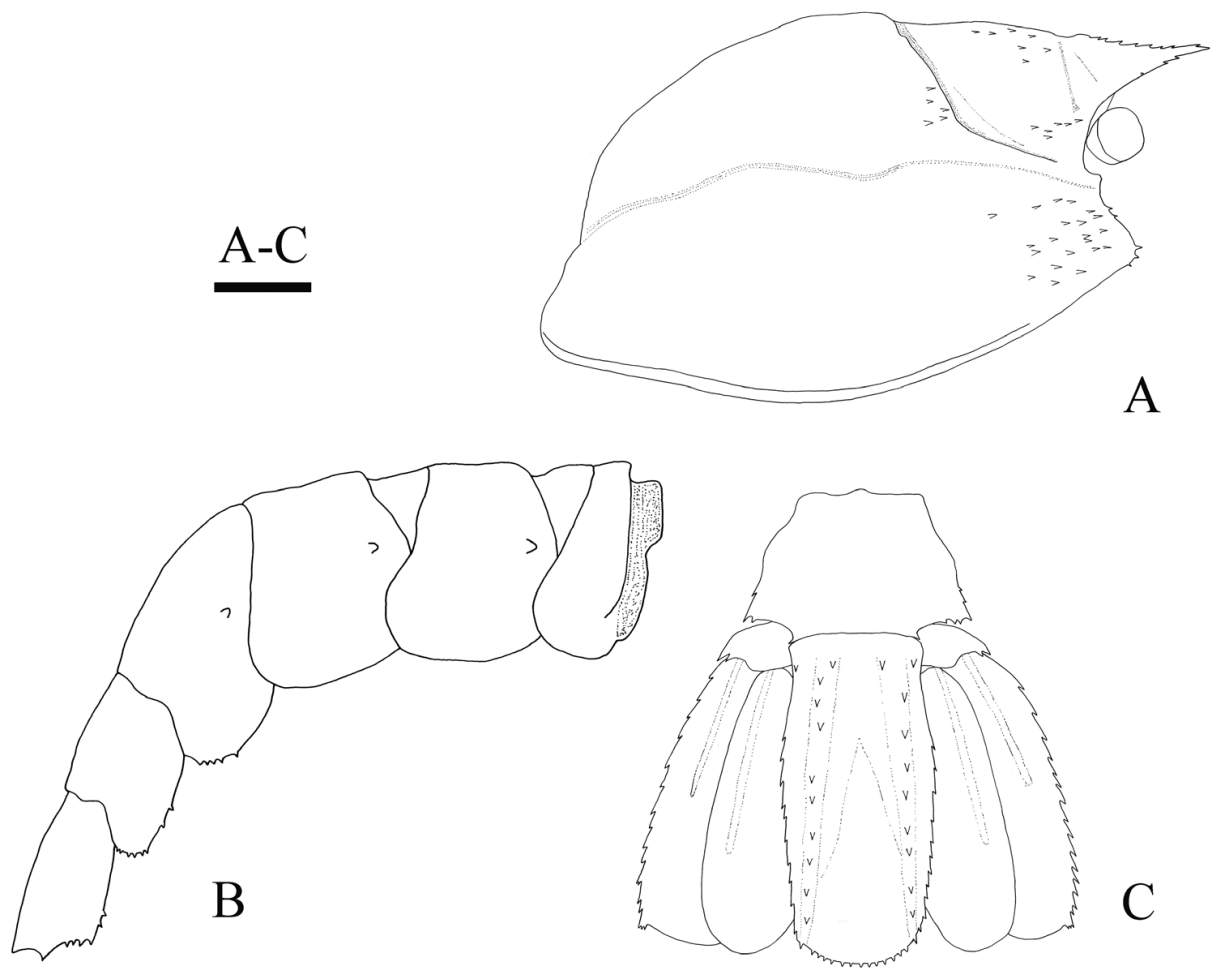
*Spongicoloides weijiaensis* sp. n. Holotype female: **A** carapace, lateral view **B** pleon, lateral view **C** telson and uropods, dorsal view. Scale bar 2 mm.

*Rostrum* (Fig. 2A) nearly horizontal, 0.26 times as long as carapace, reaching to distal margin of basal article of antennular peduncle; dorsal margin armed with eight small teeth; ventral margin armed with two small teeth on distal half; rostral base triangular in dorsal view, each ventrolateral ridge armed with a minute spine.

*Carapace* (Fig. 2A) fairly inflated; cervical groove distinct. Antennal spine blunt. Anterolateral margin with two branchiostegal spines and three (right) or five (left) pterygostomial spines; several spinules situated posterior to them. Postorbital region armed with one short longitudinal row of spinules; groups of similar spinules also present on posterior portion of cervical groove and rostrum.

Sixth *thoracic sternite* (Fig. 5A) with paired triangular plates, ventral surface concave. Seventh and eighth sternites with bilobed prominences, ventral surface concave.

*Pleomeres* (Fig. 2B) glabrous. First to third pleura broadly rounded, unarmed, and setose on ventral margin. Second to fourth pleura each with one articular knob. First somite short, divided in two sections by distinct transverse carina; posterior section of pleuron rounded. Second and third somites with shallow transverse grooves on terga. Fourth pleura with five (right) or seven (left) minute posteroventral teeth. Fifth pleura with eight (right) or nine (left) minute posteroventral teeth. Sixth pleura with six (right) or three (left) minute posteroventral teeth; posterolateral process terminating acutely.

*Telson* (Fig. 2C) quadrangular, almost twice as long as broad, slightly constricted near base, with two conspicuous dorsolateral carinae, each armed with nine (right) or ten (left) posteriorly directed spines; constricted part of each lateral margin with a single proximal submarginal spine; lateral marginal spines distinct. Setiferous posterior margin broadly rounded, with thirteen spines.

**Figure 3.**
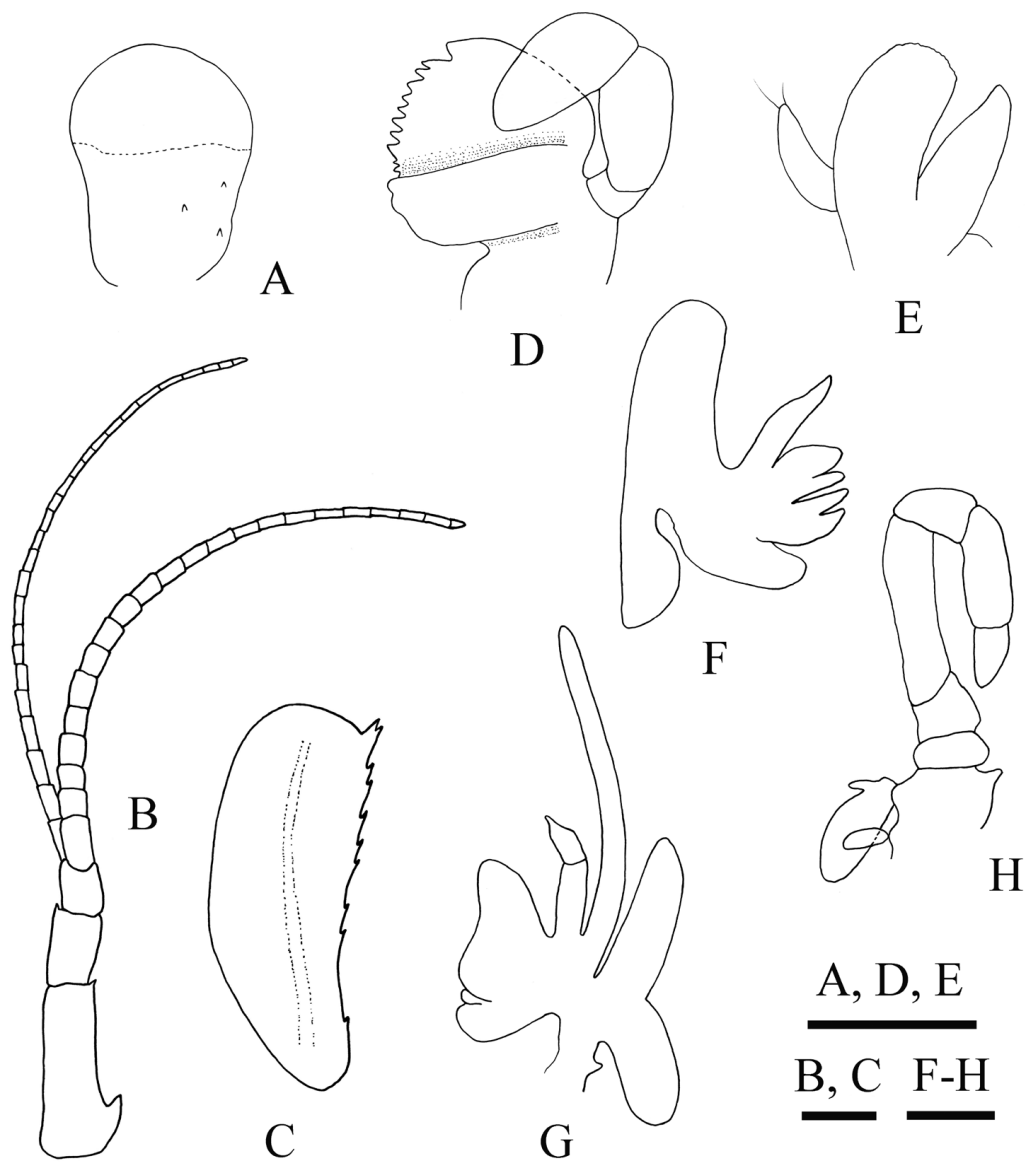
*Spongicoloides weijiaensis* sp. n. Holotype female: **A** right eye, outer view **B** right antennule, dorsal view **C** right antennal scale, dorsal view **D** right mandible, inner view **E** right maxillule, outer view **F** right maxilla, outer view **G** left first maxilliped, outer view **H** left second maxilliped, inner view. Setae omitted. Scale bars 1 mm.

*Eyes* (Fig. 3A) moderate in size; cornea globular, devoid of dark pigment, broader than eyestalk. Eyestalk armed with three minute spines.

*Antennular peduncle* (Fig. 3B) reaching mid-length of antennal scale; first article distinctly longer than both distal articles combined, with a blunt spine distolaterally; stylocerite small, subacutely pointed; second article longer than distal article, bearing a single distal spinule on inner margin; distal article unarmed.

*Antenna* with stout basicerite, bearing four (right) or three (left) large spines at distolateral angle, additional four (right) or three (left) small spines on ventrodistal margin, and two (right) or three (left) small spines on ventral surface proximally. Carpocerite overreaching first article of antennular peduncle. Antennal scale (Fig. 3C) broad; twice as long as rostrum, 2.7 times as long as wide; lateral margin slightly concave, not setiferous, with ten (right) or twelve (left) spines; distolateral bifid spine slightly falling short of or just reaching rounded distal margin of lamella; inner margin convex, Both inner and distal margins with long setae; dorsal surface with single longitudinal carina. Basal article of antennal peduncle armed with three (right) or two (left) terminal spines laterally.

*Mandible* (Fig. 3D) with 3-jointed palp; distal article oval, subequal in length to intermediate article; molar and incisor processes separated.

*Maxillule* (Fig. 3E) with simple palp bearing a pair of terminal setae; distal endite broad, its mesial margin straight; proximal endite suboval, tapering distally.

*Maxilla* (Fig. 3F) with palp tapering distally; distal and proximal endites both deeply bilobed; scaphognathite well developed.

First *maxilliped* (Fig. 3G) with bi-jointed palp; proximal article broad, 1.5 times of distal article in length; distal endite large, rounded anteriorly; proximal endite bilobed; exopod well developed; epipod large, subequally bilobed.

Second maxilliped (Fig. 3H) with endopod composed of seven articles; dactylus triangular, approximately 1.5 times as long as broad; propodus subquadrate, nearly 1.9 times of dactylus in length; carpus short, widening distally, 0.6 times as long as propodus; merus long, 2.3 times as long as carpus; ischium not fused with basis, 0.2 times as long as merus; epipod oval, with rudimentary podobranch; exopod absent.

**Figure 4.**
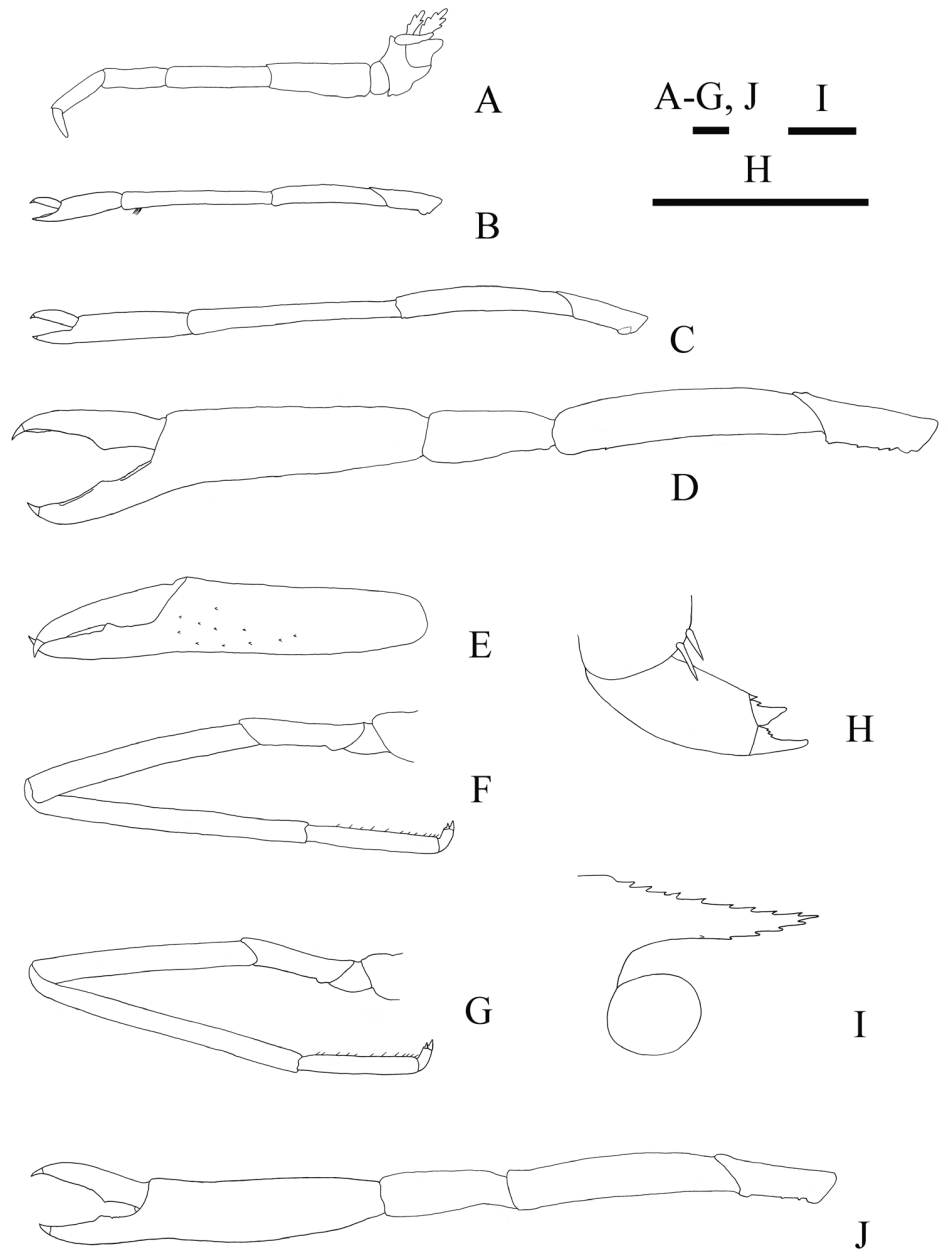
*Spongicoloides weijiaensis* sp. n. Holotype female: **A** left third maxilliped, lateral view **B** left first pereiopod, lateral view **C** left second pereiopod, lateral view **D** left third pereiopod, lateral view **E** flexor margin of right third pereiopod chela, lateral view **F** left fourth pereiopod, lateral view **G** left fifth pereiopod, lateral view **H** dactylus of left fourth pereiopod, lateral view. Paratype male: **I** rostrum, lateral view **J** left third pereiopod, lateral view. Scale bars 1 mm.

Third maxilliped (Fig. 4A) with 7-jointed endopod, slender, slightly overreaching mid-length of antennal scale in full extension; dactylus tapering distally; propodus 1.8 times as long as dactylus; carpus 1.1 times of propodal length; merus 1.7 times of carpal length; ischium subequal to merus; basis shortest, approximately 0.2 times length of ischium; coxa with epipod; exopod absent.

First *pereiopod* (Fig. 4B) reaching distal margin of antennal scale; dactylus half as long as palm; palm subcylindrical, with some grooming setae; carpus longest, nearly 2.4 times as long as palm, distal part of flexor margin of carpus with tuft of grooming setae; merus 0.7 times as long as carpus; ischium 0.5 times as long as merus; coxa and basis short, unarmed.

Second pereiopod (Fig. 4C) generally similar in shape to first pereiopod, longer, overreaching distal margin of antennal scale by length of chela; dactylus 0.4 times as long as palm; carpus 1.9 times as long as palm; merus 0.8 times as long as carpus; ischium 0.4 times as long as merus; coxa and basis short, unarmed.

Third pereiopod (Fig. 4D) strongest and longest, overreaching distal margin of antennal scale by length of chela. Fingers terminating each in strongly curved, corneous claw, tips crossing; fixed finger with deep longitudinal concavity proximally, bearing single rounded tooth at nearly mid-length of cutting edge and with short row of small teeth on proximal cutting edge, distoventral margin without row of teeth; dactylus 0.6 times of palm length, protruded at proximal 0.4 of length, with concavity on distal half portion; palm almost equal to merus in length, subcylindrical; some minute teeth present on distal half of flexor margin of propodus (Fig. 4E); carpus widening distally, nearly half as long as palm; merus of right third pereiopod unarmed; merus of left third pereiopod bearing minute tooth at approximately distal 0.2 of its length on flexor margin; ischium 0.9 times as long as carpus, flexor margin with row of 3–4 small teeth, distolateral margin also with similar teeth; basis and coxa short, unarmed.

Fourth and fifth pereiopods similar, moderately long and slender. Fourth pereiopod (Fig. 4F) overreaching distal margin of antennal scale by length of dactylus and propodus; dactylus (Fig. 4H) short, compressed laterally, biunguiculate primarily, ventral unguis shorter than dorsal unguis, both clearly demarcated, with some much smaller accessory teeth arising from bases of both ventral and dorsal ungues; propodus approximately 0.4 times length of carpus, armed with single row of eleven or twelve movable spines on flexor margin; carpus longest; merus 0.8 times length of carpus; ischium half-length of merus, unarmed; coxa and basis short and stout.

Fifth pereiopod (Fig. 4G) overreaching distal margin of antennal scale by dactylus and half-length of propodus; propodus 0.4 times length of carpus, armed with single row of twelve or thirteen movable spines on flexor margin; merus 0.7 times length of carpus; ischium 0.4 times length of merus, unarmed; coxa and basis short and stout.

All pereiopods with small and blunt protrusions on proximal parts of ischial flexor margins.

**Figure 5.**
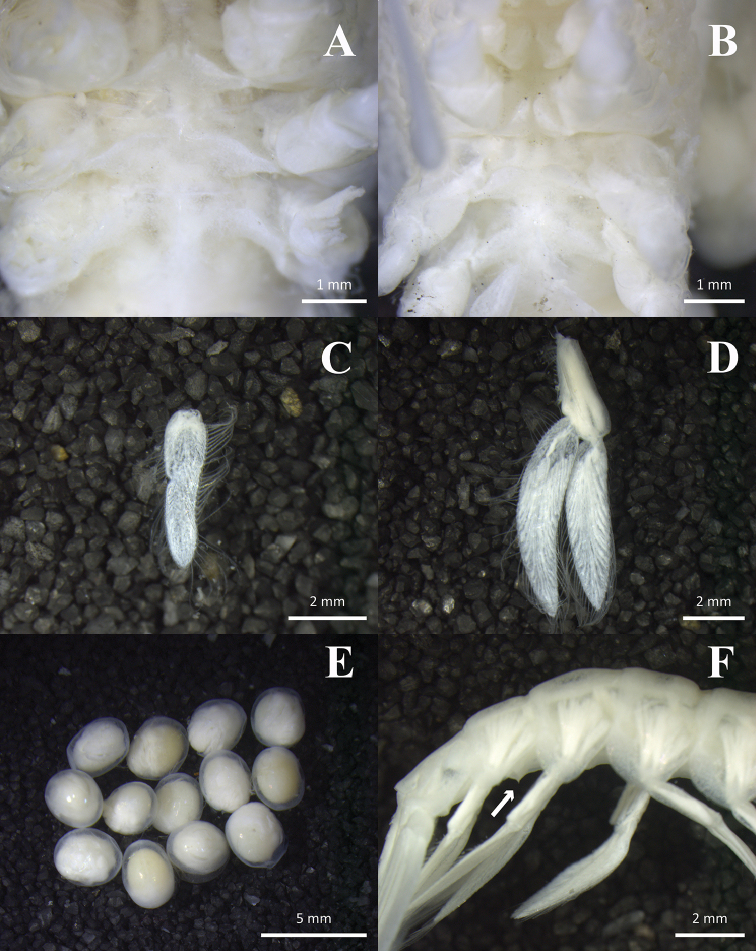
*Spongicoloides weijiaensis* sp. n. Holotype female: **A** thoracic sternites, ventral view **C** first pleopod, lateral view **D** second pleopod, lateral view **E** eggs, outer view. Paratype male: **B** thoracic sternites, ventral view **F** second to sixth pleonites, showing spine of fifth sternite, lateral view.

First *pleopod* (Fig. 5C) smallest, uniramous. Second to fifth pleopods biramous. Second pleopod (Fig. 5D) with protopod shorter than rami, bearing ovipositing setae on dorsal and ventral margins; mesial surface with ridge bearing ovipositing setae. Third to fifth pleopods generally similar, decreasing in size posteriorly; fourth and fifth pleopods lacking ovipositing setae.

Uropod (Fig. 2C) with stout protopod; lateral margin terminating in two spines. Endopod and exopod each with single weak dorsal carina. Lateral margin of exopod slightly convex with row of fourteen (left) or fifteen (right) acute teeth, excluding broad trilobed tooth on distolateral angle. Endopod ovate, falling short of posterior margin of telson.

Holotype female carrying thirteen eggs (Fig. 5E). Pleonal sternites unarmed. Branchial formula summarized in Table 1.

**Table 1.** Branchial formula of *Spongicoloides weijiaensis* sp. n. (r = rudimentary).

	Maxillipeds			Pereiopods				
	I	II	III	I	II	III	IV	V
Pleurobranchs	0	0	1	1	1	1	1	1
Arthrobranchs	r	1	2	2	2	2	2	0
Podobranchs	0	r	0	0	0	0	0	0
Epipods	1	1	1	0	0	0	0	0
Exopods	1	0	0	0	0	0	0	0

###### Main characters of paratype male.

Rostrum (Fig. 4I) nearly horizontal, reaching to distal margin of basal article of antennular peduncle, dorsal margin armed with nine small teeth; ventral margin armed with four small teeth on distal half; rostral base triangular in dorsal view, each ventrolateral ridge armed with a minute spine. Thoracic sternites (Fig. 5B) much narrower than in female; sixth thoracic sternite lacking paired distinct spines anteromesially. Fifth pleonal sternite (Fig. 5F) with one spine. Dorsolateral carinae of telson conspicuous, armed with seven (right) or eight (left) posteriorly directed spines. Left antennal scale with bifid distolateral spine, right antennal scale with simple distolateral spine. Fingers of third pereiopod (Fig. 4J) relatively shorter than in female, 0.3 times length of chela; fixed finger without row of small teeth on distoventral margin; ischium flexor margin with two small teeth. Dactyli of fourth and fifth pereiopods biunguiculate, ventral unguis shorter than dorsal unguis, bearing some much smaller accessory teeth. Distolateral angles of uropodal exopods with single blunt tooth (possibly abraded) or with bifid tooth.

###### Etymology.

The specific name, *weijiaensis*, refers to the type locality, the Weijia Guyot, part of the Magellan Seamount Chain in the northwestern Pacific.

###### Color in life.

Body whitish, translucent; corneas, some intrathoracic organs and eggs pale yellow.

###### GenBank accession numbers.

KY404237 (16S rRNA), KY404238 (COI).

###### Discussion.

One of the most important taxonomic features of species assigned to *Spongicoloides* is the branchial formula. Although some gills are rather fragile and easily detachable structures, and their development may be variable (rudimentary, simple or well-developed), the total number of gills is still one of the first and main characters to examine when one is dealing with *Spongicoloides*.

*Spongicoloides weijiaensis* sp. n. shares the presence of two arthrobranchs on the third maxilliped and first through fourth pereiopod with *Spongicoloides novaezelandiae* Baba, 1979 from Chatham Rise east of New Zealand, *S. hawaiiensis* Baba, 1983 from Hawaii, and *S. iheyaensis* Saito, Tsuchida & Yamamoto, 2006 from southern Japan (Baba 1979, 1983, Saito et al. 2006).

The other five species of *Spongicoloides* can be distinguished in having substantial differences in the gill formulae, such as the third maxilliped bears a single arthrobranch in *S. evolutus* (Bouvier, 1905a) and the first through fourth pereiopod each bear a single arthrobranch in *S. galapagensis* Goy, 1980, *S. inermis* (Bouvier, 1905b), *S. profundus* Hansen, 1908 and *S. tabachnicki* Burukovsky, 2009.

The new species can be separated from *S. novaezelandiae* by the fourth pleuron bearing several minute teeth on the posteroventral margin (vs fourth pleuron broadly rounded in *S. novaezelandiae*); the flexor margin of the third pereiopod ischium armed with a row of 2-4 small teeth (vs unarmed in *S. novaezelandiae*); the second maxilliped with a single arthrobranch (vs with paired arthrobranchs in *S. novaezelandiae*); and the fourth and fifth pereiopod dactyli with accessory teeth at the bases of the ungues (vs absent or at most with small angle in *S. novaezelandiae*) (cf. Baba 1979).

The new species can be also distinguished from *S. hawaiiensis*, e.g. by the carapace bearing groups of spinules posterior to the rostrum, orbits, cervical groove and pterygostomian angle (vs almost spineless in *S. hawaiiensis*); the more numerous spines on the lateral and posterior margins of the telson and the lateral margin of the antennal scale; the flexor margin of the third pereiopod ischium armed with some small teeth (vs unarmed in *S. hawaiiensis*); and the dorsal surface of the antennal scale and uropodal exopod each with a single mesial or submesial longitudinal carina (vs with two longitudinal carinae in *S. hawaiiensis*) (cf. Baba 1983).

*Spongicoloides weijiaensis* sp. n. differs from *S. iheyaensis* by the sixth pleonite unarmed dorsally (vs armed with one spine or a longitudinal row of small spines on dorsal midline in *S. iheyaensis*); the fixed finger of the third pereiopod unarmed on distoventral margin (vs bearing a short row of small teeth on the distoventral margin in *S. iheyaensis*); the third pereiopod ischium armed with a row of 2-4 small teeth on flexor margin (vs unarmed in *S. iheyaensis*); the dorsal surface of the antennal scale and uropodal exopod with one mesial or submesial longitudinal carina (vs with two longitudinal carinae in *S. iheyaensis*); and the ovigerous female with much smaller number of eggs (13 eggs in holotype female of *S. weijiaensis* sp. n. vs 229 eggs in holotype female of *S. iheyaensis*) (cf. Saito et al. 2006).

#### Key to the known Pacific species of *Spongicoloides*

**Table:** 

1	Third maxilliped and first through fourth pereiopod each with single arthrobranch; propodus and ischium of third maxilliped armed with spines on mesial margins; dorsal median carina of uropodal endopod with a single spine	***S. galapagensis* Goy, 1980**
–	Third maxilliped and first through fourth pereiopod each with two arthrobranchs; propodus and ischium of third maxilliped unarmed on mesial margins; dorsal median carina of uropodal endopod unarmed	**2**
2	Sixth pleonite armed with one spine or longitudinal row of small spines on midline; fixed finger of third pereiopod armed with several (3-9) teeth on distoventral margin	***S. iheyaensis* Saito, Tsuchida & Yamamoto, 2006**
–	Sixth pleonite unarmed on midline; fixed finger of third pereiopod unarmed on distoventral margin	**3**
3	Carapace with spinules on postrostral and postorbital regions; third pereiopod ischium with row of 2-4 small teeth on flexor margin	***S. weijiaensis* sp. n.**
–	Carapace without spinules on postrostral and postorbital regions; flexor margin of third pereiopod ischium unarmed	**4**
4	Carapace with scattered spinules on anterolateral region; third pereiopod ischium with prominent process on distoventral margin; posterior margin of telson with eight spines	***S. novaezelandiae* Baba, 1979**
–	Carapace without scattered spinules on anterolateral region, third pereiopod ischium without distoventral process; posterior margin of telson with three spines	***S. hawaiiensis* Baba, 1983**

## Supplementary Material

XML Treatment for Spongicoloides weijiaensis
